# Supplementary material for: Membrane and luminal proteins reach the apicoplast by different trafficking pathways in the malaria parasite Plasmodium falciparum
Source: PeerJ. 2017 Apr 27;5:e3128. doi: 10.7717/peerj.3128 (PMC5410153; doi:10.7717/peerj.3128)

**Membrane and luminal proteins reach the apicoplast by different trafficking pathways in the malaria parasite *Plasmodium falciparum***

Rahul Chaudhari, Vishakha Dey, Aishwarya Narayan, Shobhona Sharma, Swati Patankar

**Supplementary Images showing raw data for the blots for Figure 1C, Figure 6A and Figure 6E**

**We reproducibly find single bands on Western blots with the antibodies against PfTPx_Gl_ (Chaudhari et al, 2012 and subsequent experiments done in the lab) and only pre-processed and processed proteins for ACP-GFP using anti-GFP antibodies. No bands are developed by the antibodies in any other parts of the blot. Therefore, a few of these blots were cut before development so as to save on antibodies and because two different antibodies had to be used on different parts of the same blot.**

Supplementary Image 1: Blot for ACP-GFP panel in Figure 1C

Supplementary Image 2: Blot for PfTPx_Gl_ panel in Figure 1C

Supplementary Figure 3: Loading Control in Figure 1C

Supplementary Image 4: Blot for ACP-GFP panel in Figure 6A

Supplementary Image 5: Blot for PfTPx_Gl_ panel in Figure 6A

Supplementary Image 6: Blot for ACP-GFP panel in Figure 6E

Supplementary Image 7: Blot for PfTPx_Gl_ panel in Figure 6E

Supplementary Image 1


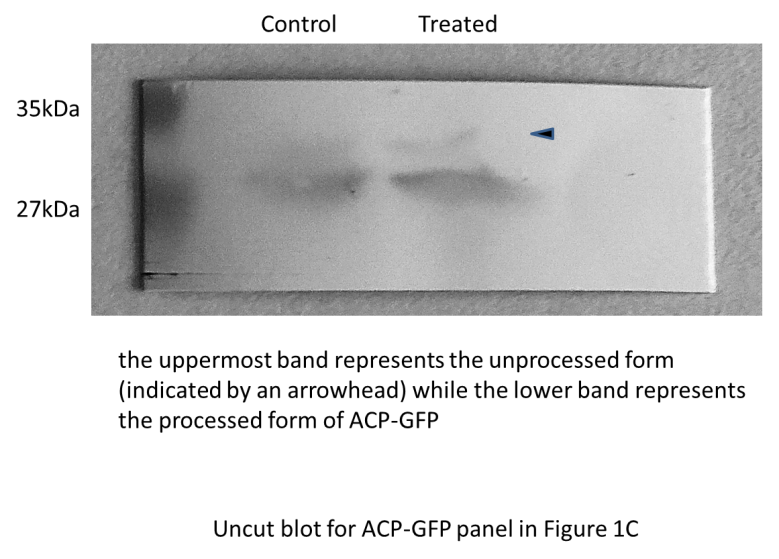


Supplementary Image 2


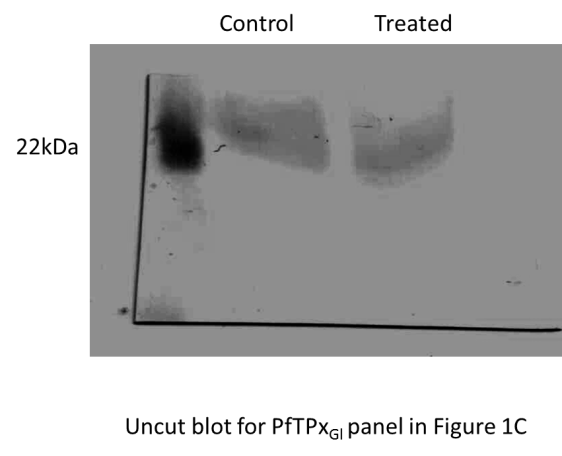


Supplementary image 3


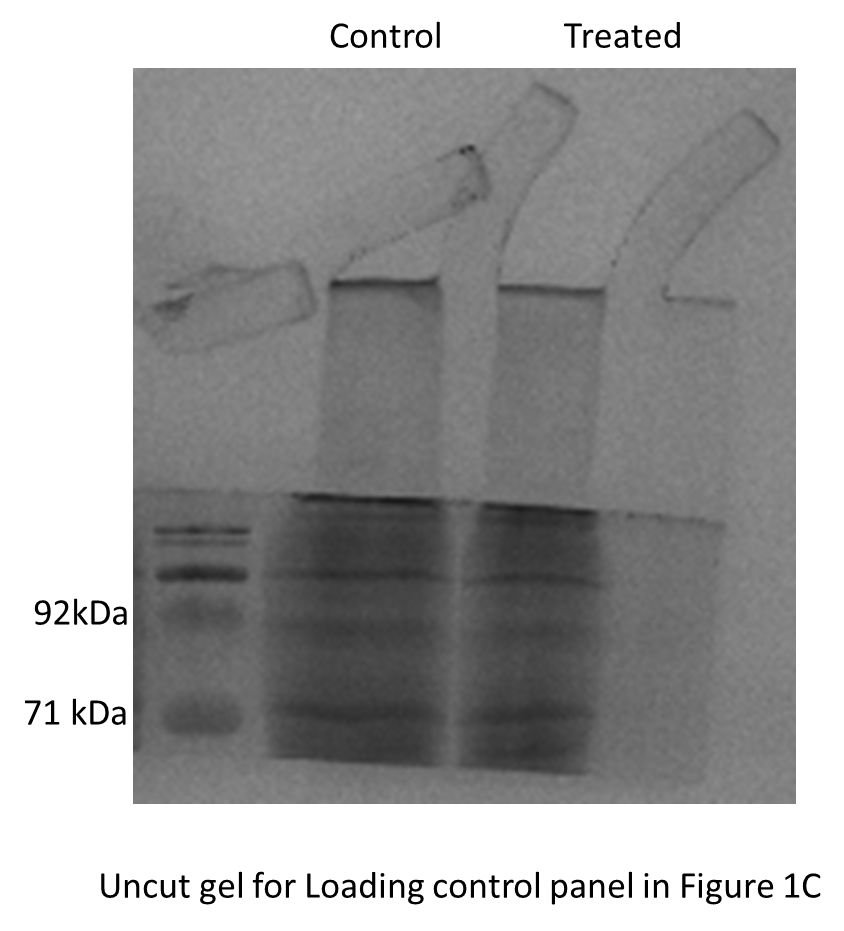


Supplementary Image 4


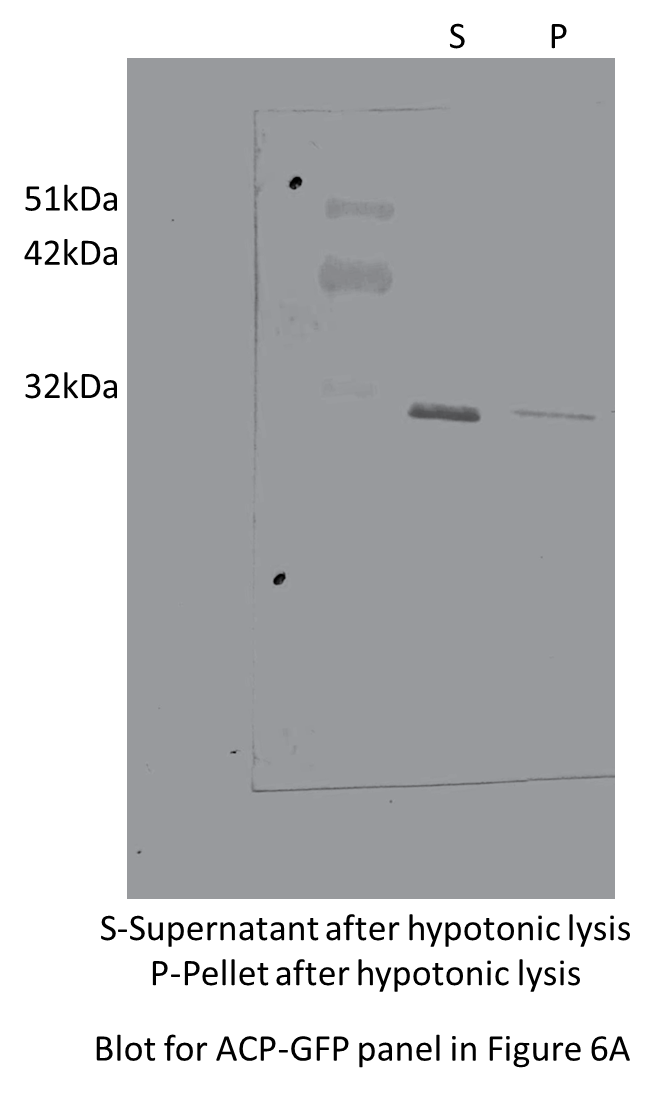


Supplementary Image 5


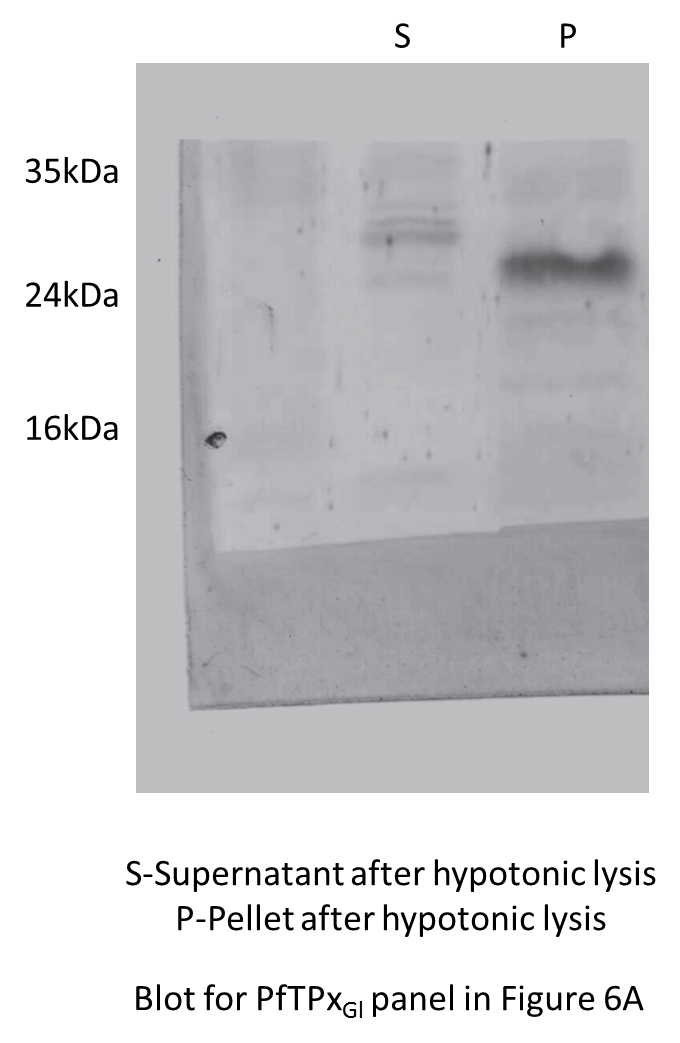


Supplementary Image 6


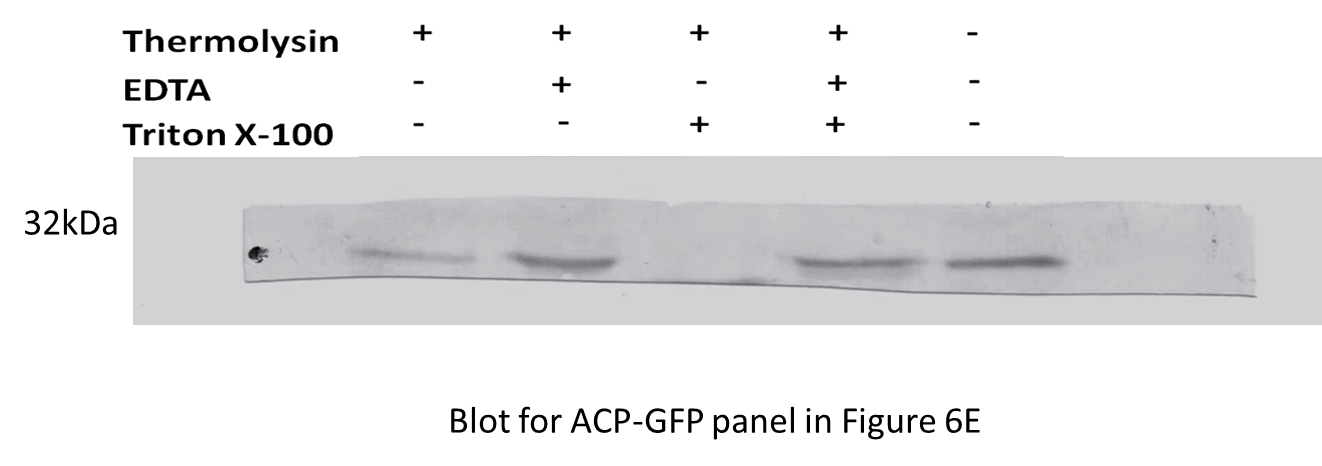


Supplementary Image 7


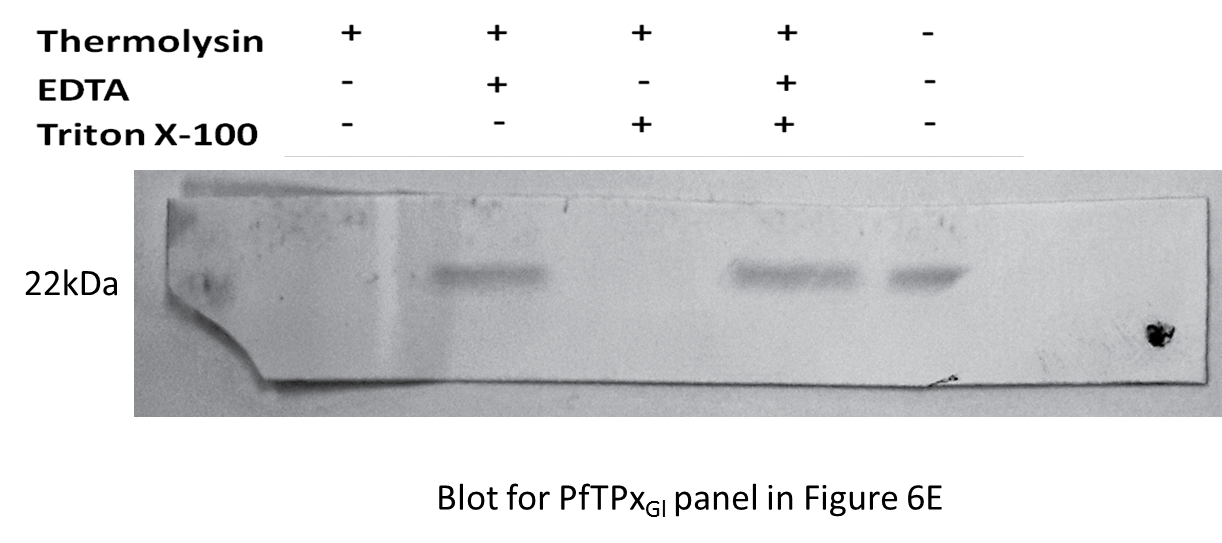

Supplement: Supplemental Information 1 [file peerj-05-3128-s001.docx]
