# Supplementary material for: Membrane and luminal proteins reach the apicoplast by different trafficking pathways in the malaria parasite Plasmodium falciparum
Source: PeerJ. 2017 Apr 27;5:e3128. doi: 10.7717/peerj.3128 (PMC5410153; doi:10.7717/peerj.3128)
Supplement: Table S1 [file peerj-05-3128-s002.docx]

| **Protein** | **Primary antibody used (Dilution)** | **Reference for the antibody used** | **Method of antibodies generation** | **Method of checking the specificity of antibodies** | **Secondary antibody used (Dilution)** |
| --- | --- | --- | --- | --- | --- |
| PfTPx_Gl_ | rabbit raised anti-PfTPx_Gl_ (1:250) | [Chaudhari et al. 2012](#_ENREF_6) and this study | Polyclonal antibodies generated against full length protein | Western blot of the parasite lysates, Immunoprecipitation of the parasite lysates | Goat anti-Rabbit IgG (H+L) Alexa Fluor® 568 (Invitrogen™) (1:1000) / Goat anti-Rabbit IgG (H+L) Alexa Fluor® 488 (Invitrogen™) |
| ACP | rabbit raised anti-ACP (1:250) | This study | Polyclonal antibodies generated against the synthetic peptide corresponding to the amino acids from 41-55 of the ACP protein sequence | Western blot of the parasite lysates | Goat anti-Rabbit IgG (H+L) Alexa Fluor® 568 (Invitrogen™) (1:1000) |
| UROD | mouse raised anti-UROD (1:250) | [Nagaraj et al. 2009](#_ENREF_31) | Protein-A purified polyclonal IgG antibodies generated against UROD protein lacking N-terminal 43 amino acids, | Western blot of the parasite lysates | Goat anti-Mouse IgG (H+L) Alexa Fluor® 568 (Invitrogen™) (1:1000) |
| PfFC | mouse raised anti-FC (1:250) | Varadharajan et al. 2004 | Polyclonal antibodies generated against FC protein lacking first 113 amino acids | Western blot of the parasite lysates, Immunoprecipitation of the parasite lysates | Goat anti-Mouse IgG (H+L) Alexa Fluor® 568 (Invitrogen™) (1:1000) |
| BiP | mouse raised anti-BiP (1:100) | [Siddiqui et al. 2013](#_ENREF_33), Kaderi Kibria et al. 2015 | Polyclonal antibodies generated against a synthetic peptide corresponding to the last 11 carboxy terminus amino acids of PfBip conjugated with KLH (Thermo Fishcher Scientific) | Western blot and immunoprecipitation of the parasite lysates (Author communication) | Goat anti-Mouse IgG (H+L) Alexa Fluor® 568 (Invitrogen™) (1:1000) |
| GRASP | mouse raised anti-GRASP (1:100) | [Siddiqui et al. 2013](#_ENREF_33), Kaderi Kibria et al. 2015 | Polyclonal antibodies generated against a synthetic peptide corresponding to the 14 amino acids from 529-543 of PfGRASP conjugated with KLH (Thermo Fishcher Scientific) | Western blot and immunoprecipitation of the parasite lysates (Author communication) | Goat anti-Mouse IgG (H+L) Alexa Fluor® 568 (Invitrogen™) (1:1000) |
| EMP1 | rabbit raised anti-EMP1 (1:500) | Ganguly et al. 2015 | Polyclonal antibodies generated against 290 amino acids VARC (cytoplasmic domain) region from the EMP1 protein sequence | Western blot of the parasite lysates (Author communication) | Goat anti-Rabbit IgG (H+L) Alexa Fluor® 568 (Invitrogen™) (1:1000) |
| KAHRP | mouse raised anti-KAHRP (1:150) | Ganguly et al. 2015 | Polyclonal antibodies generated against 49 amino acids K2A1(central lysine rich region) region from the KAHRP protein sequence | Western blot of the parasite lysates (Author communication) | Goat anti-Mouse IgG (H+L) Alexa Fluor® 650 (Invitrogen™) (1:400) |
| GFP | mouse raised anti-GFP (1:250) | Roche Life Sciences | Mixture of two monoclonal antibodies, both clones are purified Mouse IgG_1ĸ_ | Western blot of the wild type and mutant GFP proteins | Goat anti-Mouse IgG (H+L) Alexa Fluor® 568 (Invitrogen™) (1:1000) |
