# Supplementary material for: Membrane and luminal proteins reach the apicoplast by different trafficking pathways in the malaria parasite Plasmodium falciparum
Source: PeerJ. 2017 Apr 27;5:e3128. doi: 10.7717/peerj.3128 (PMC5410153; doi:10.7717/peerj.3128)
Supplement: Table S2 — S2 (A): Percentage distribution of PfTPxGl in control, drug treated and drug washed out parasites. (B): Percentage of parasites showing intact/collapsed ER-Golgi morphology in drug treated and drug washed out cells. (C): Percentage of parasites showing PfEMP1 and KAHRP distribution in drug treated and drug washed out parasites. [file peerj-05-3128-s003.docx]

Table S2A : Percentage distribution of PfTPx_Gl_ in control, drug treated and drug washed out parasites

|  | Parasites showing apicoplast localization of PfTPx_Gl_ | Parasites showing mitochondrial localization of PfTPx_Gl_ | Parasites showing cytosolic localization of PfTPx_Gl_ | Parasites showing disrupted localization after treatments | Total number of parasites observed | % of parasites showing apicoplast localization of PfTPx_Gl_ | % of parasites showing mitochondrial localization of PfTPx_Gl_ | % of parasites showing cytosolic localization of PfTPx_Gl_ | % inhibition of apicoplast trafficking after treatments |
| --- | --- | --- | --- | --- | --- | --- | --- | --- | --- |
| Control D10-ACP_leader_-GFP parasites  (Figure 1) | 18 | 14 | 1 |  | 33 | 54.5% | 42.4% | 3% |  |
| D10-ACP_leader_-GFP parasites treated with AlF_4_^-^ (Figure 1) | 2 |  |  | 113 | 115 |  |  |  | 98% |
| D10-ACP_leader_-GFP parasites treated with vinblastine  (Figure 2) | 2 |  |  | 31 | 33 |  |  |  | 94% |
| D10-ACP_leader_-GFP parasites treated with nocodazole  (Supplementary Figure S4) | 1 |  |  | 35 | 36 |  |  |  | 97% |
| Vinblastine washed out D10-ACP_leader_-GFP parasites  (Figure 3) | 11 | 12 |  |  | 23 | 47.8% | 52.2% |  |  |
| Nocodazole washed out D10-ACP_leader_-GFP parasites  (Supplementary Figure S4) | 10 | 12 |  |  | 22 | 45% | 55% |  |  |
| Control 3D7 parasites  (Supplementary Figure S3) | 11 | 6 |  |  | 17 | 64.7% | 35.9% |  |  |
| 3D7 parasites treated with AlF_4_^-^  (Supplementary Figure S3) | 6 |  |  | 66 | 72 |  |  |  | 92% |
| 3D7 parasites treated with vinblastine  (Supplementary Figure S3) | 1 |  |  | 32 | 33 |  |  |  | 96% |
| 3D7 parasites treated with nocodazole  (Supplementary Figure S3) | 1 |  |  | 34 | 35 |  |  |  | 97% |
| Vinblastine washed out 3D7 parasites  (Supplementary Figure S3) | 25 | 23 |  |  | 48 | 52% | 48% |  |  |
| Nocodazole washed out 3D7 parasites  (Supplementary Figure S3) | 29 | 30 |  |  | 59 | 49% | 51% |  |  |

Table S2B : Percentage of parasites showing intact/collapsed ER-Golgi morphology in drug treated and drug washed out cells

|  | Parasites showing intact endoplasmic reticulum | Parasites showing intact Golgi | Parasites showing dispersed endoplasmic reticulum | Parasites showing dispersed Golgi | Total number of parasites observed | % parasites showing dispersal |
| --- | --- | --- | --- | --- | --- | --- |
| D10-ACP_leader_-GFP parasites treated with AlF_4_^-^ (Figure 4) | 14 |  | 0 |  | 14 |  |
| D10-ACP_leader_-GFP parasites treated with vinblastine (Figure 4) | 26 |  | 0 |  | 26 |  |
| Vinblastine washed out D10-ACP_leader_-GFP parasites (Figure 4) | 27 |  | 0 |  | 27 |  |
| D10-ACP_leader_-GFP parasites treated with AlF_4_^-^ (Figure 5) |  | 1 |  | 16 | 17 | 95% |
| D10-ACP_leader_-GFP parasites treated with vinblastine (Figure 5) |  | 1 |  | 17 | 18 | 95% |
| Vinblastine washed out D10-ACP_leader_-GFP parasites (Figure 5) |  | 10 |  | 1 | 11 | 90% Reversion |

Table S2C : Percentage of parasites showing PfEMP1 and KAHRP distribution in drug treated and drug washed out parasites

|  | Parasites showing trafficking of PfEMP1 to RBC cytosol and surface | Parasites showing trafficking of KAHRP to RBC cytosol and surface | Parasites showing disrupted PfEMP1 trafficking to the RBC cytosol and surface | Parasites showing disrupted KAHRP trafficking to the RBC cytosol and surface | Total number of parasites observed | % inhibition of the trafficking to the RBC cytosol and surface |
| --- | --- | --- | --- | --- | --- | --- |
| D10-ACP_leader_-GFP parasites treated with AlF_4_^-^ (Figure 1) | 5 |  | 142 |  | 147 | 96% |
| D10-ACP_leader_-GFP parasites treated with vinblastine (Figure 2) | 4 |  | 130 |  | 134 | 97% |
| D10-ACP_leader_-GFP parasites treated with AlF_4_^-^ (Supplementary Figure S2) |  | 3 |  | 87 | 90 | 97% |
| D10-ACP_leader_-GFP parasites treated with vinblastine (Supplementary Figure S2) |  | 1 |  | 56 |  | 98% |
