# Supplementary figures and images for: Membrane and luminal proteins reach the apicoplast by different trafficking pathways in the malaria parasite Plasmodium falciparum
Source: PeerJ. 2017 Apr 27;5:e3128. doi: 10.7717/peerj.3128 (PMC5410153; doi:10.7717/peerj.3128)

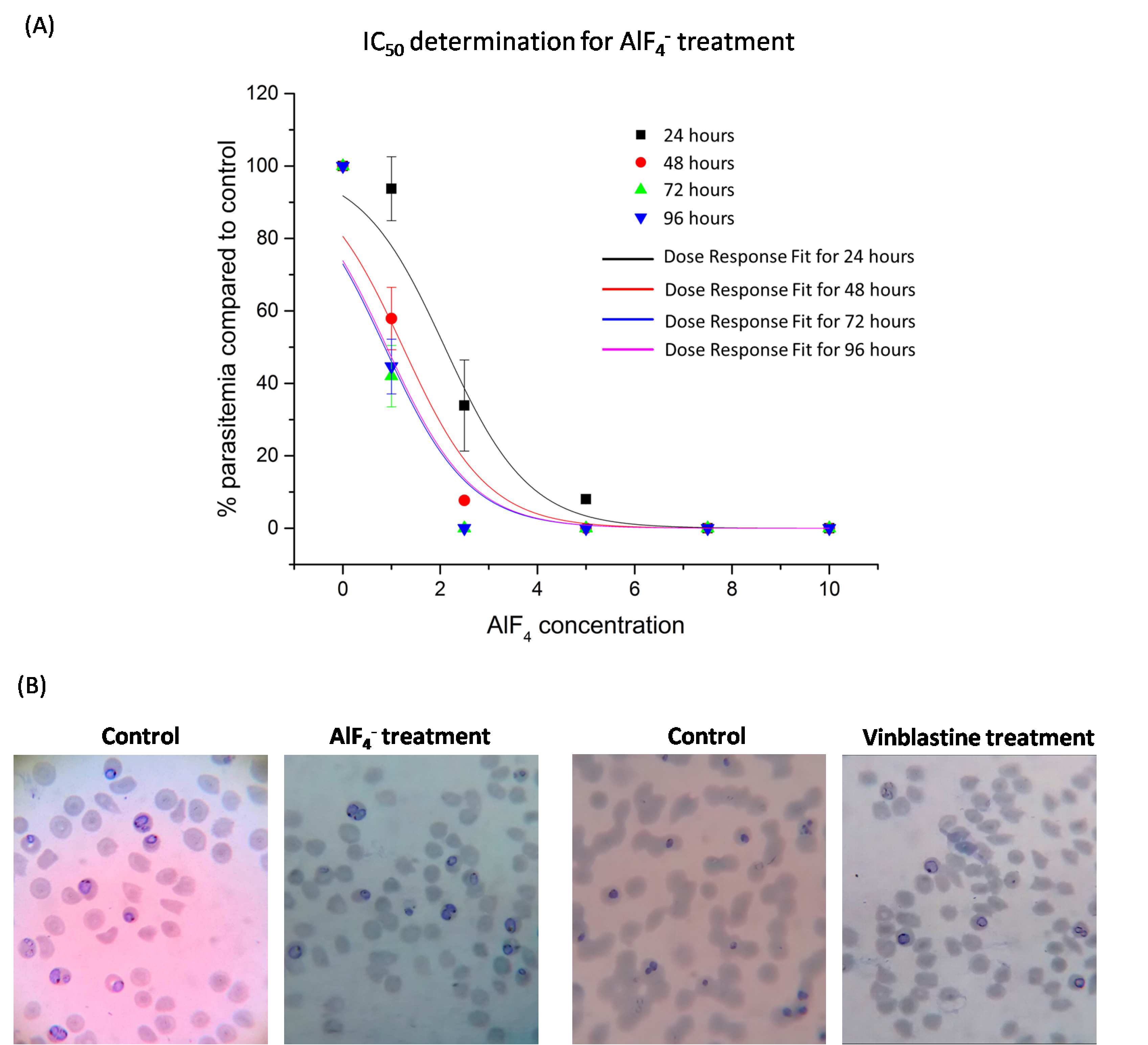

Supplement: Figure S1 — (A) Dose response curve fit for P. falciparum treated at different concentrations of Aluminum tetrafluoride (AlF4−). IC50 value calculated non-linear regression of the sigmoidal dose response equation from OriginPro was found to be 1.23 ±0.16 µM. 95% confidence interval was found to be ± 0.16. Note that no parasite survival was observed at higher AlF4− concentrations of 7.5 and 10 µM at 24 hours. (B) Images showing a normal morphology of P. falciparum treated with Aluminum tetrafluoride (AlF4−) and vineblastine at IC50 concentration of 1.2 µM and 100 nM respectively for 18 ±2 hours. [file peerj-05-3128-s004.png]

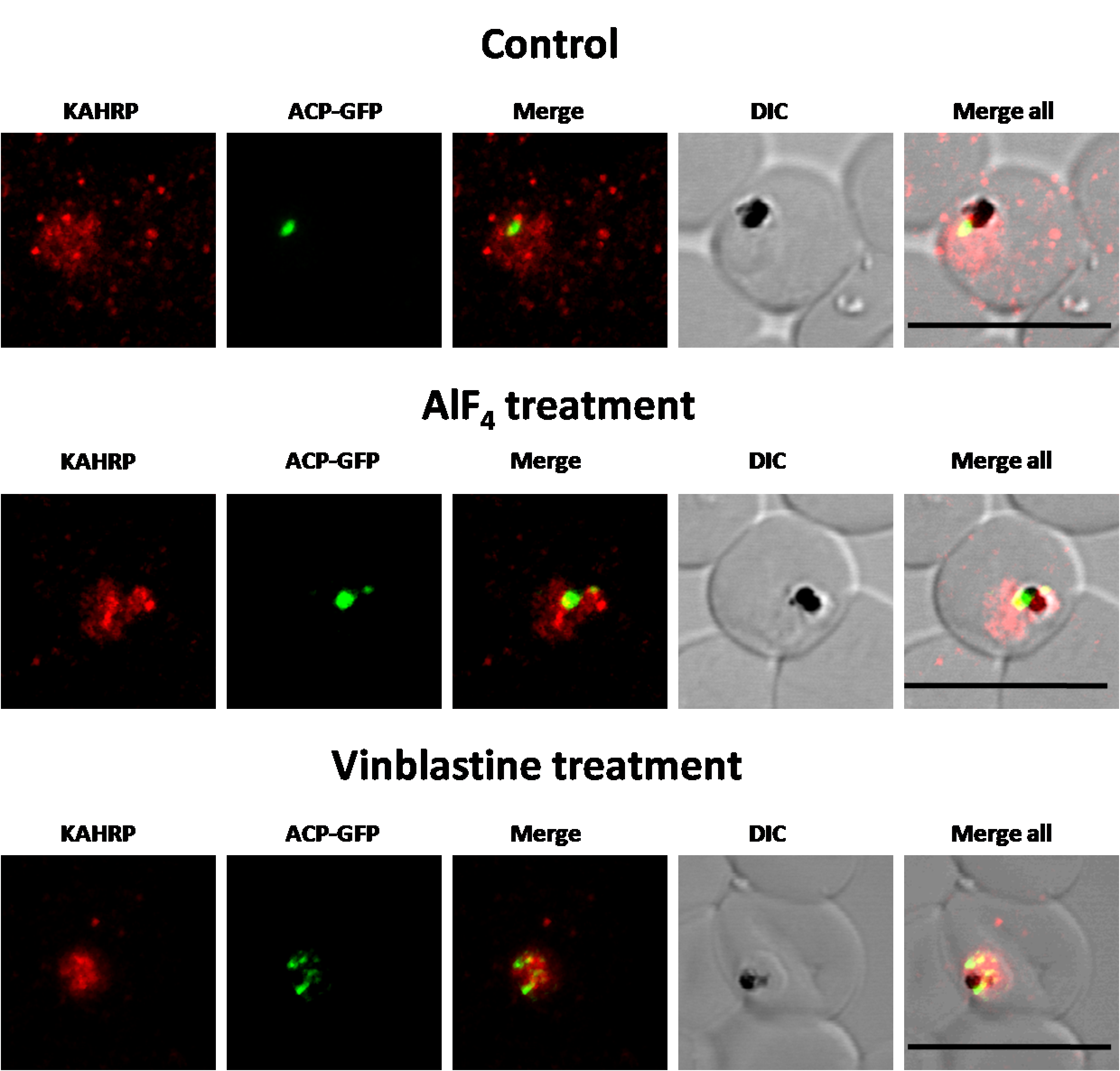

Supplement: Figure S2 — In AlF4− treatment, KAHRP trafficking to the RBC cytosol and surface was inhibited in 97% of the parasites 87 parasites analyzed. In vinblastine treated cultures, KAHRP trafficking to the RBC cytosol and surface was inhibited in 98% of the 56 parasites analyzed (See Table S2 for quantification). Scale Bar: 10 µm. [file peerj-05-3128-s005.png]

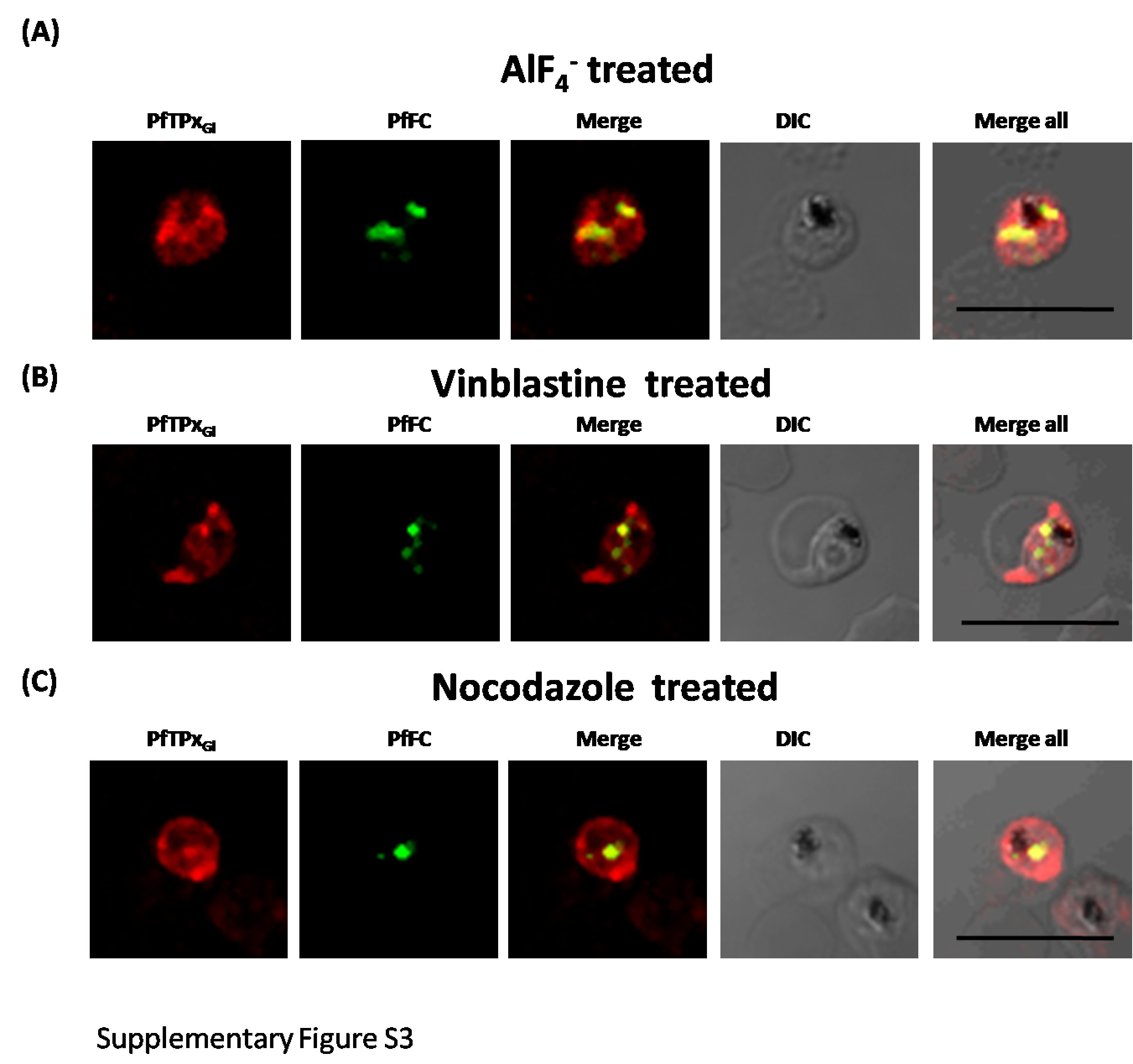

Supplement: Figure S3 — (A) PfTPxGl and PfFC co-localization in AlF4−-treated parasites, (B) PfTPxGl and PfFC co-localization in vinblastine-treated parasites, (C) PfTPxGl and PfFC co-localization in nocodazole-treated parasites. In this experiment, PfTPxGl was found to be co-localized with the mitochondrial marker protein PfFC in 40% of the treated parasites suggesting that trafficking of PfTPxGl to the mitochondrion may be partially disrupted by the treatments. Scale Bar: 10 µm. [file peerj-05-3128-s006.png]

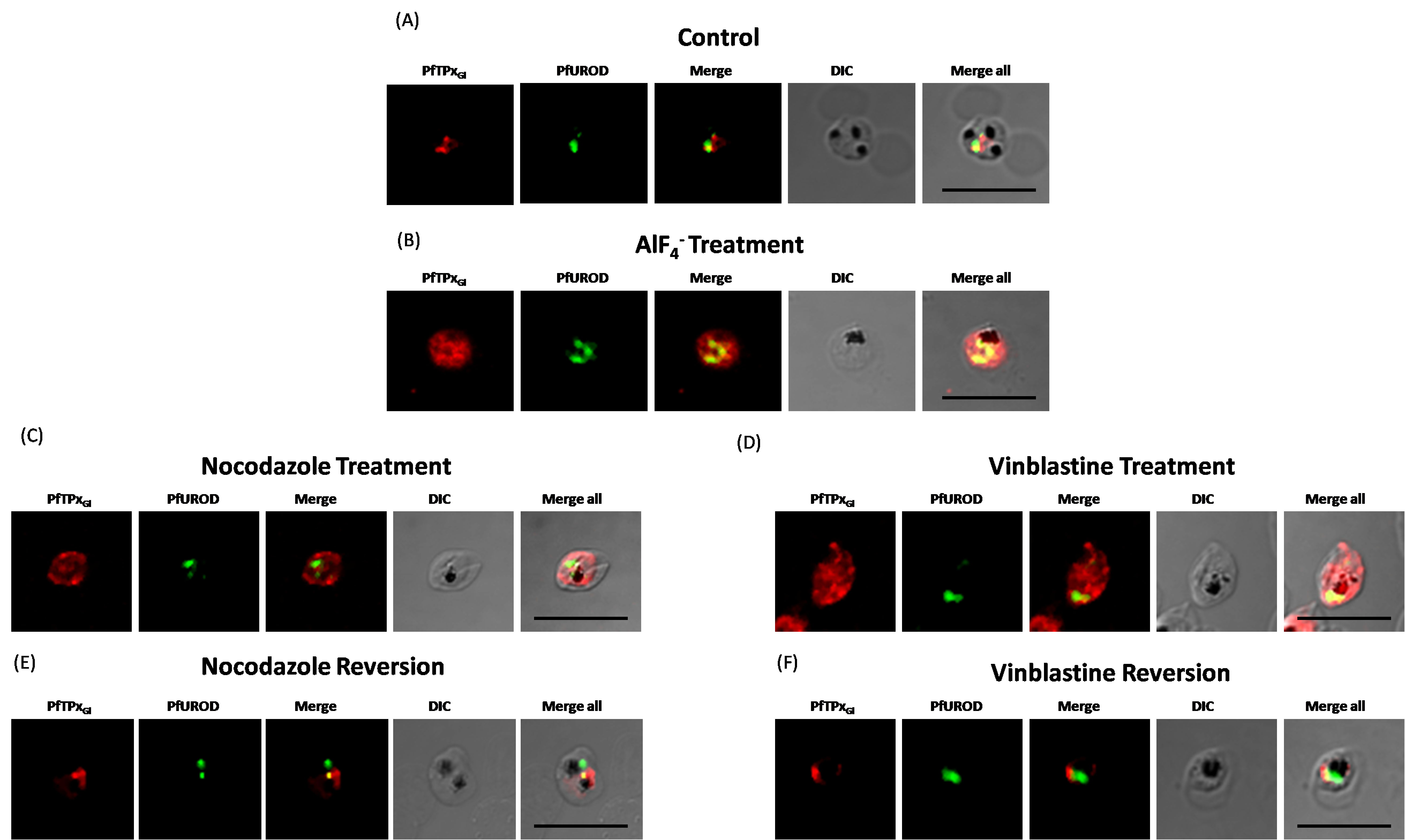

Supplement: Figure S4 — (A) PfTPxGl localization in solvent control parasites, (B) PfTPxGl localization in AlF4−-treated parasites, (C) PfTPxGl localization in nocodazole-treated parasites, (D) PfTPxGl localization in vinblastine-treated parasites, (E) PfTPxGl localization in parasites reverted after nocodazole treatment, (F) PfTPxGl localization in parasites reverted after vinblastine treatment. PfTPxGl targeting was disrupted in 97% (35 parasites counted) of nocodazole-treated parasites and in 96% (33 parasites counted) of vinblastine-treated parasites. For AlF4−-treatment, 92% of the 72 parasites analyzed showed disrupted PfTPxGl signal. In reversion experiments, localization of PfTPxGl in vinblastine washed out parasites was reverted to the apicoplast in 52% parasites (48 parasites counted), while in nocodazole washed out parasites 49% showed apicoplast localization (59 parasites counted) (See Table S2 for quantification). Scale Bar: 10 µm. [file peerj-05-3128-s007.png]

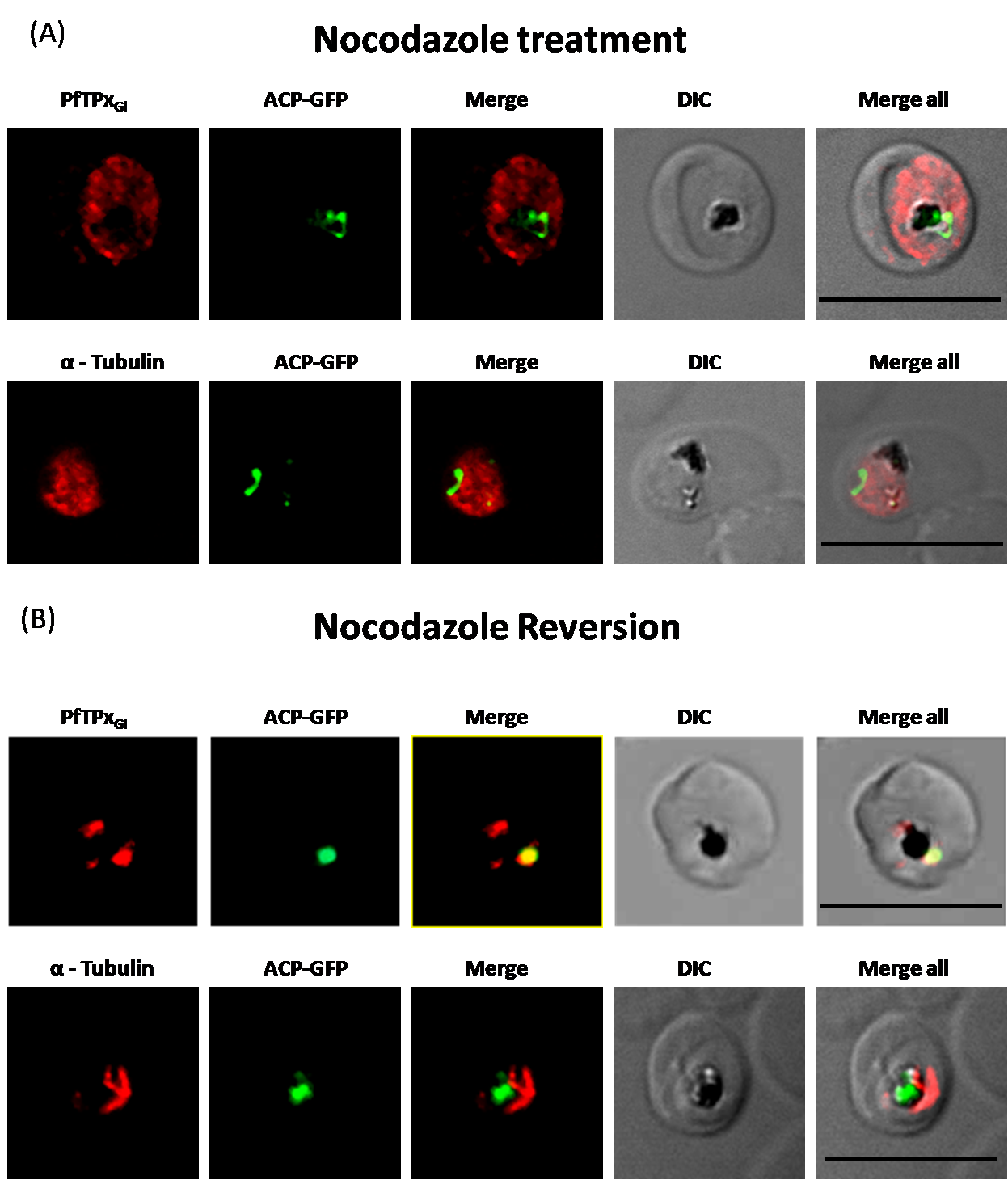

Supplement: Figure S5 — (A) In these experiments, targeting to the apicoplast was inhibited in 97% of the parasites with nocodazole treatment (35 parasites counted), (B) Immunofluorescence images showing PfTPxGl and microtubules in D10-ACPleader-GFP parasites with drug washed out. Reversion of PfTPxGl localization to the organelles and intact microtubular structures observed in parasites in drug washed out medium after nocodazole treatment. In reversion experiment, localization of PfTPxGl in nocodazole washed out parasites was reverted to the apicoplast in 45% parasites (22 parasites counted). Scale Bar: 10 µm. [file peerj-05-3128-s008.png]

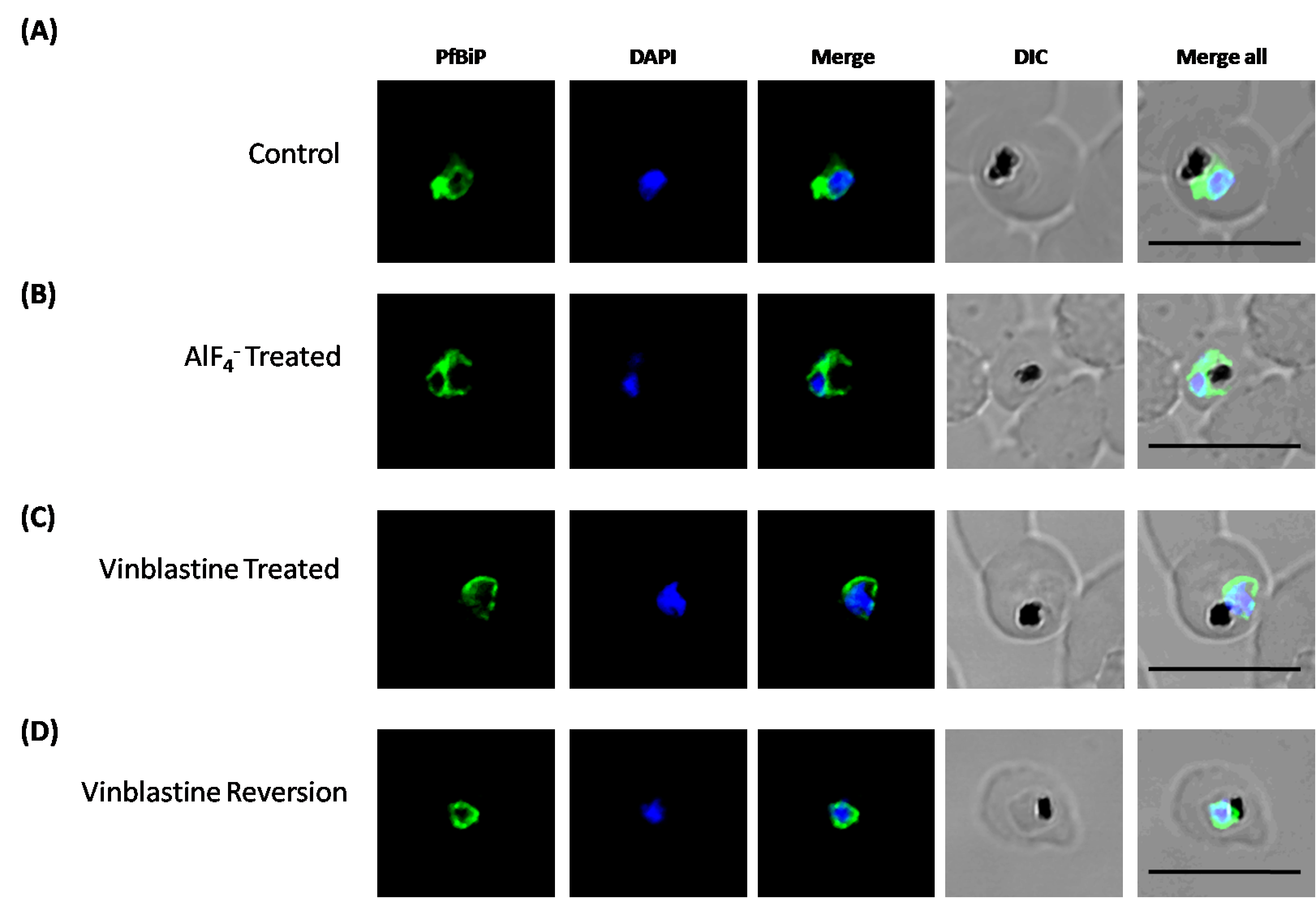

Supplement: Figure S6 — (A) PfBiP localization in control parasites, (B) ER morphology in AlF4−- treated parasites (C) ER morphology in vinblastine-treated parasites, (D) ER morphology in parasites reverted after vinblastine treatment. Scale Bar: 10 µm. [file peerj-05-3128-s009.png]

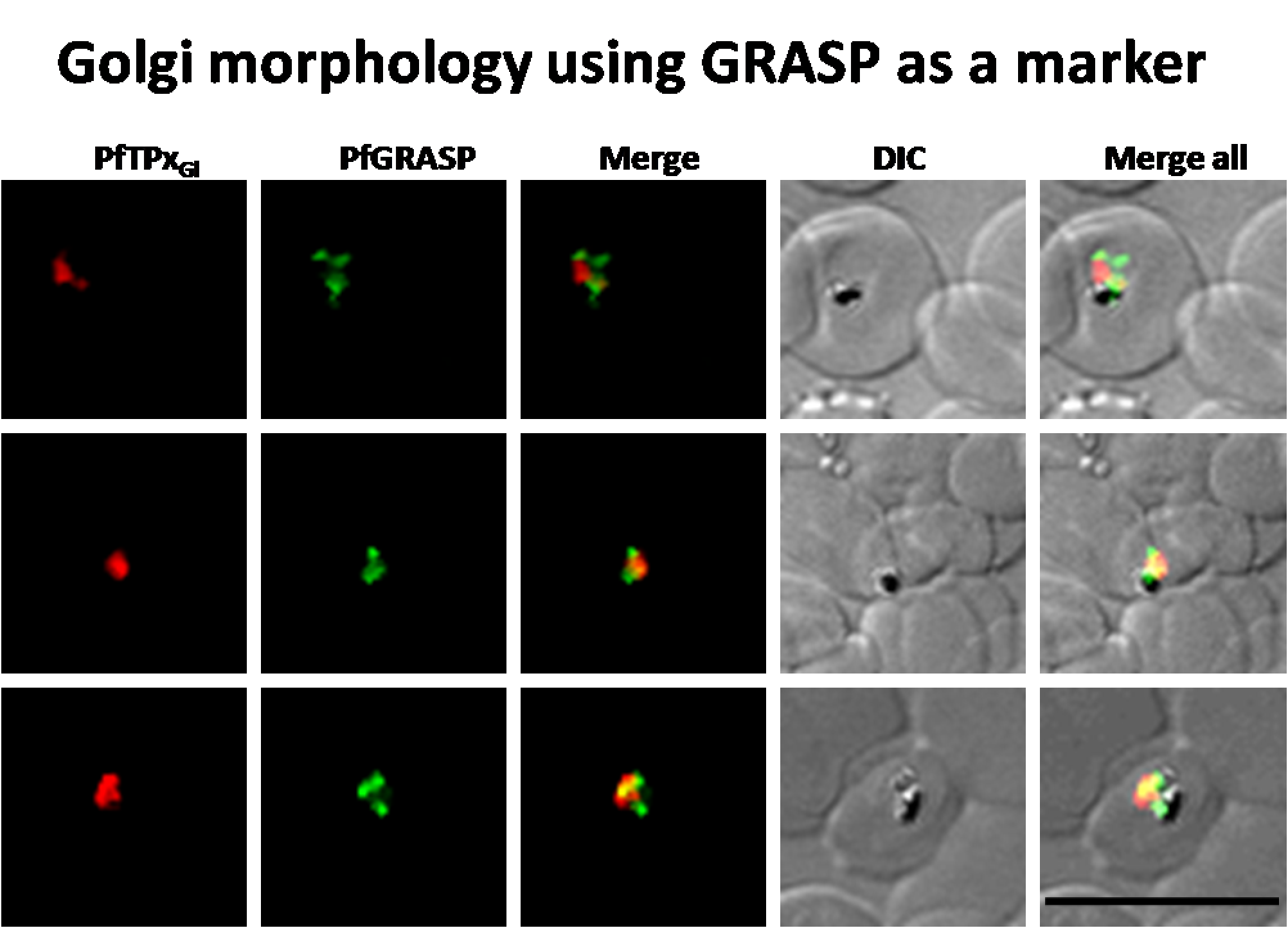

Supplement: Figure S7 [file peerj-05-3128-s010.png]
